# Procedure and Clinical Success of Drug-Coated Balloon Fistuloplasty of the Drainage Vein in Dysfunctional Native Arteriovenous Fistulas

**DOI:** 10.1155/2021/8266673

**Published:** 2021-12-28

**Authors:** Zeki Temiztürk, Davut Azboy, Fevzi Sarper Türker

**Affiliations:** Elazığ Fethi Sekin City Hospital, Cardiovascular Surgery Clinic, Elazığ, Turkey

## Abstract

**Purpose:**

Native arteriovenous fistulas (AVFs) are the most effective vascular access (VA) for haemodialysis. We aimed to evaluate the results of balloon angioplasty (fistuloplasty) from drainage vein performed for the treatment of AVF dysfunction in haemodialysis patients and examine potential patient and AVF-associated factors that might affect such results.

**Methods:**

This is a nonrandomized, retrospective, and single-centred study. A total of 105 balloon fistuloplasties were performed for dysfunctional AVFs of 82 haemodialysis patients. Patients were treated with a drug-coated balloon according to standard procedures. Evaluations were performed by physical examinations and if needed by color Doppler imaging in every 6 months. The primary endpoint was patency by balloon fistuloplasty. Patency was evaluated clinically by detecting the thrill in AVF and by the adequacy of the dialysis. Multidimensional scaling (MDS) technique was used as a method for the statistical analysis.

**Results:**

The success of the procedure after the first attempt was 85.3% with 70 patients. Patency in the 6th, 12th, 18th, and 24th months were 63 (76.8%), 60 (73.1%), 53 (64.6%), and 44 (54%), respectively. The procedure was considered successful when the thrill was detected in AVF and when dialysis was adequate. The statistical analysis by MDS revealed that patients' age was the most effective factor acting on the procedure success followed by the age of AVF. Other patient-associated and AVF-associated factors were not found as effective statistical evaluation.

**Conclusions:**

Haemodialysis through native AVFs with restored functionality contributes positively to the life span and the quality of life of the patient. Probably, advanced age and high fistula age are unfavourable factors leading to the development of neointimal hyperplasia and venous stenosis. Balloon fistuloplasty of the draining vein is an effective and safe method regardless of patient age and the age of AVF.

## 1. Introduction

Adequate vascular access (VA) is absolutely necessary for haemodialysis. VA has been considered both a vital necessity and an iatrogenically induced weak spot for the proper conduct of haemodialysis therapy [1]. No major advances have occurred in establishing and maintaining VA in the last three decades and VA dysfunction remains to be a major cause of morbidity in haemodialysis patients. It has been estimated that VA dysfunction is responsible for 20% of all hospitalizations in the haemodialysis population [2]. VA dysfunction is closely related to mortality and morbidity in haemodialysis patients. It has long been suggested that there is a need for interventions associated with low restenosis rates [3]. It is universally agreed that native arteriovenous fistulas (AVF) are superior to synthetic arteriovenous grafts (AVGs) and tunnelled central venous catheters for haemodialysis access. However, AVFs and AVGs have limited lifespans [3]. One-year patency rates in the US are 68% for AVFs and 49% for AVGs. Although such rates can be as high as 83% in Europe, there is still need for further improvement [4].

The standard treatment of stenosis caused by neointimal hyperplasia is balloon angioplasty. Despite the simplicity and practicality of the method, conventional balloons may become inadequate for the treatment of haemodialysis access stenosis [5]. Although the blood flow is restored by the intraluminal expansion of the balloon, the integrity of the neointimal tissue is disrupted leading to an increased risk for venous neointimal hyperplasia. This is a healing response to the trauma caused by the endovascular intervention and may cause further stenosis. This risk is well recognized for AVG anastomoses and in stenotic mature fistulas [2, 6, 7]. Drug-coated balloons (DCBs) have been introduced as an alternative to conventional balloon angioplasty. DCBs inhibit neointima formation locally reducing intimal hyperplasia and the risk of stenosis in coronary and peripheral vessels by delivering antiproliferative agents, such as paclitaxel, directly to the lesion. However, current data on the efficacy of DCBs for maintaining VA in intimal-hyperplasia-associated stenoses are inadequate [8, 9].

In order to determine the prevalence of chronic kidney disease (CKD) in Turkey, Gültekin et al. evaluated a total of 10,872 participants (mean age 40.5 ± 16.3 years; 55.7% women) in their study in 2011. The overall prevalence of CKD was found to be 15.7% with a higher frequency in women compared to men (18.4% vs. 12.8%, *P* < 0.001) and the prevalence increased with increasing age of participants. The same study reported that cardiovascular risk factors were more prevalent in CKD patients [10]. That study emphasizes that the prevalence of CKD is high in the Turkish population, and hence, the need for haemodialysis and VA is also high. Another emphasis is that the prevalence of CKD is high in old age and cardiovascular risk factors are common in these patients. In this study, we aimed to investigate patient and AVF-associated factors that might affect the efficacy, safety, and patency rates of balloon fistuloplasty of the draining vein in dysfunctional AVFs in haemodialysis patients.

## 2. Methods

### 2.1. Study Population

This retrospective, single-centred study was conducted in the cardiovascular surgery clinic of Elazığ Fethi Sekin City Hospital in the period between August 2015 and September 2019. The study included 82 patients (mean age 59.05 ± 9.3 range 27–88 years) with AVFs that were created for haemodialysis due to end-stage renal failure and that became dysfunctional in time. All patients in whom native AVFs were created in our clinic or in an external centre for VA for haemodialysis, in whom the thrill could not be detected during active haemodialysis, and in whom haemodialysis could not be performed adequately were included in the study. Age, the anastomotic site in the arm, or location and form of stenosis were not defined as exclusion criteria. All fistulas were mature native AVFs. Of the study patients, 50 were men and 32 were women. AVFs for haemodialysis were created in the right and the left arms of 16 and 66 patients, respectively. Of the AVFs, 55 were radio-cephalic, 24 were brachio-cephalic, and 3 were brachio-basalic. AVFs became dysfunctional due to thrombosis in 43 patients and due to stenosis in 39 patients. A total of 105 balloon fistuloplasty procedures were performed in 82 patients (twice in 19 patients and three times in 4 patients). An aneurysm was present in the AVF tract in 28 patients (Table 1). The patency of AVFs was evaluated in a period of 24 months through interviews with staff in respective haemodialysis centres and via physical examinations of patients during outpatient clinic visits for follow-up. The major parameters for patency were the adequacy of haemodialysis through the AVF as a route for VA and to detect the thrill in the AVF tract. Patients could not be followed up for various reasons, in whom a draining vein did not develop, patients with severe venous aneurysms (>5 cm), patients with skin necrosis and ulcerations at the fistula tract, patients receiving treatment for active cancers, and patients with advanced heart failure were not included in the study. The study protocol was approved by the local Ethics Committee (Decision no. 00298–2021). The study was conducted in accordance with the principles of the Declaration of Helsinki.

### 2.2. Preoperative Evaluation

**Figure 1:**
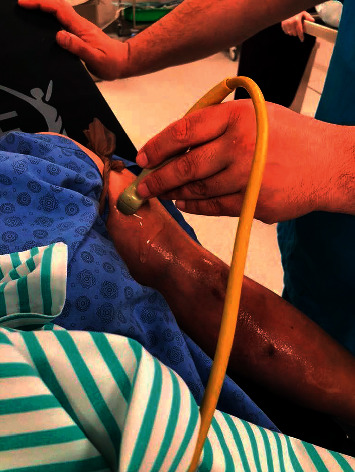
Preprocedural and intraprocedural examination by ultrasonography.

The patients were evaluated in the outpatient clinic by applying a tourniquet to observe whether a draining vein developed sufficiently. The absence of the thrill and pulsatility increased bleeding time after needle removal, swelling, or oedema and were considered the major signs of stenosis. After physical examinations, an ultrasonography (USG) examination in the outpatient clinic was performed on the AVF tract by the surgeon, who would perform the procedure (Figure 1).

USG examinations included morphological assessment in B-mode, color Doppler examinations, and Doppler USG assessments of the fistula. In USG examinations, flow velocities were measured, anastomosis lines were evaluated, and venous flow rates were calculated. A venous flow rate below 300 mL/min was considered inadequacy of AVF for haemodialysis. Such cases were prepared for the procedure. Patients were hospitalized after blood tests were performed.

### 2.3. Endovascular Procedure

Patients underwent the procedure in the angiography laboratory or in the hybrid room in the surgery suite. After cleaning the surgical site, all patients were administered local anaesthesia. To prevent bleeding and hematoma formation after the procedure, the draining vein was punctured under USG guidance by using a portable device used during surgery.

**Figure 2:**
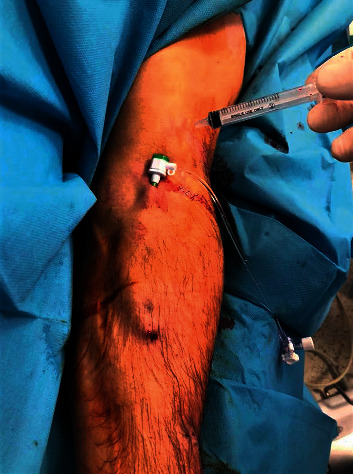
Intervention through the draining vein.

**Figure 3:**
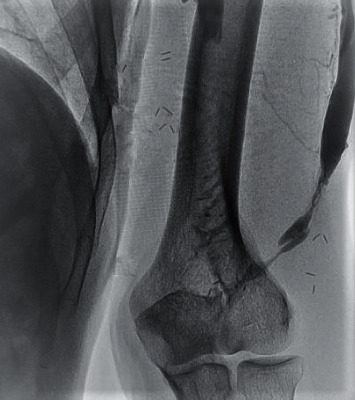
Juxta-anastomotic stenosis in AVF.

A 6F short radial sheath was inserted into the draining vein in a retrograde fashion proximally to the AVF anastomosis (Figure 2). Heparin was administered through the sheath at a dose of 5000 IU. Creating a manual occlusion proximal to the inserted sheath, the feeding artery, the anastomosis, and the draining vein were visualized by the manual administration of a radio-opaque agent directly through the sheath. The procedure was planned mainly based on the obtained images (Figure 3).

**Figure 4:**
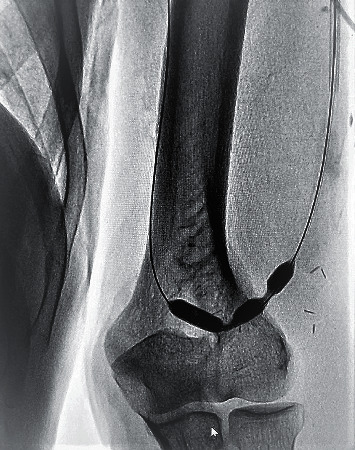
Balloon fistuloplasty on stenotic sites.

The problematic segment was crossed with a short 0.035-inch hydrophilic guide wire. The hydrophilic wire was passed through the anastomosis; it was advanced in the arterial segment, and a loop was created (Figure 4).

**Figure 5:**
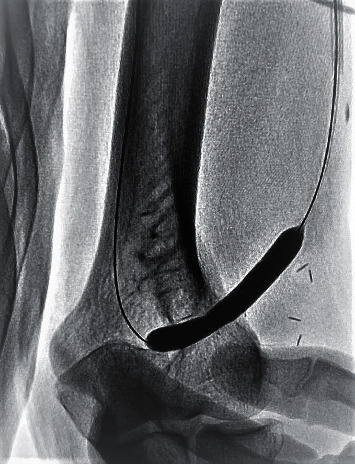
Dilation of stenotic site.

**Figure 6:**
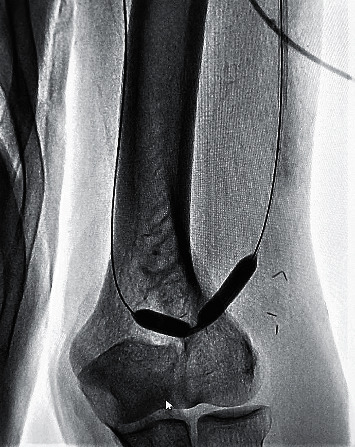
Dilation in anastomotic site.

**Figure 7:**
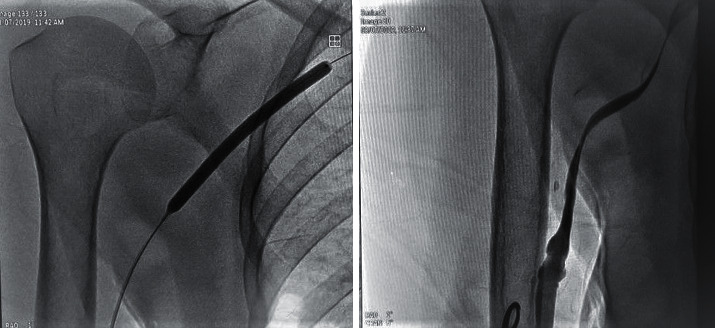
Central vein stenosis and balloon fistuloplasty.

Paclitaxel-coated endovascular balloons of 4–40 mm or 5–40 mm in size were used for stenosis close to the arterial segment and close to the anastomosis and of 5–40 mm or 6–40 mm in size and were used for draining vein stenosis (Figures 5–7).

After the termination of the stenosis, the balloon was kept inflated for 2 min to prevent elastic recoiling. When the image indicating the success of the procedure was obtained, the procedure was concluded after checking the thrill at the anastomosis. Conditionally, the pressure was increased up to 24 atmospheres for stenosis difficult to terminate. No repeat images were taken to avoid further patient exposure to radio-opaque material. After sheath removal, haemostasis was achieved by manual compression.

### 2.4. Clinical Success and Follow-Up

The clinical follow-up of the patients was performed through manual examinations and Doppler USG examinations. After the procedure, patients received enoxaparin and ASA for five days. After five days, patients were treated only with ASA. Communication was maintained with the respective haemodialysis centres that patients attended. Thus, information was obtained from the physician following up the patient haemodialysis centres. Follow-up visits were performed in the outpatient clinic. The procedure was repeated for the second and third time in some patients. Absence of thrill in AVF or inadequate dialysis was considered a clinical failure. The patency rates in the 6^th^, 12^th^, 18^th^, and 24^th^ months were determined using the clinical records of the patients and through interviews with staff at haemodialysis centres. Primary patency was defined as the time between the first intervention until access thrombosis and repeat endovascular treatment. Patency rates after the second intervention were defined as secondary patency. Patency rates after the third procedure were defined as tertiary patency. A total of 105 balloon fistuloplasty procedures were performed on 82 patients. Out of 70 patients, in whom the first procedure was successful, the procedure was performed for the second time on 19 patients and for the third time on 4 patients. No patients died. Only four patients had minor bleeding that did not require surgical intervention.

### 2.5. Statistical Analysis

**Figure 8:**
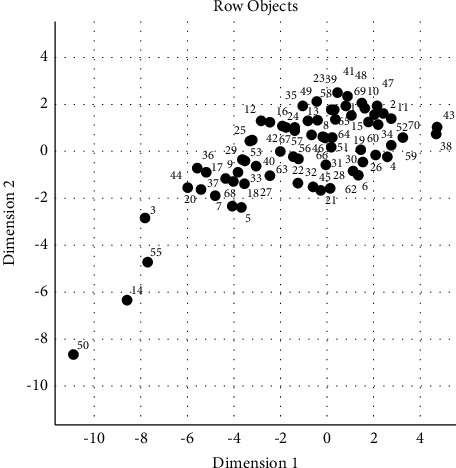
MDS map to identify similar/dissimilar patients based on evaluated characteristics.

Multidimensional scaling (MDS) is a graphical technique that is used to visualize proximities of objects in a low-dimensional space. In MDS graphs, each object or variable is represented by a point. When two points are close to each other, it is concluded that these two objects are similar or related to each other [11–14]. In this study, MDS was performed to investigate relations between patient characteristics and patency in order to classify variables and find out associations with respect to their similarities (Figure 8). Two different goodness-of-fit criteria, namely, *R*^2^, and the stress coefficient were used to determine the suitability of the MDS technique in assessing similarities of the *X*-axis (patency) and the *Y*-axis (variables).

## 3. Results

**Figure 9:**
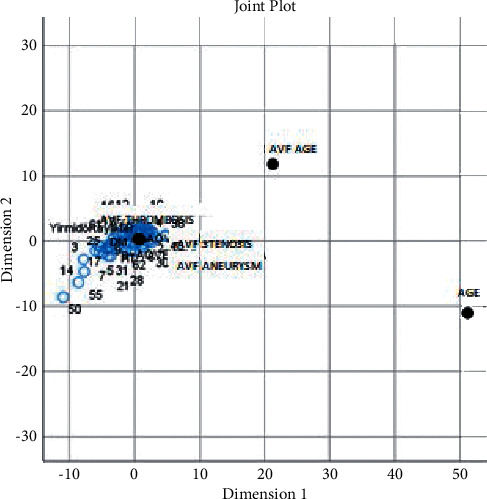
MDS map to identify similar/dissimilar characteristics of the patients.

No repeat procedure was performed on 12 out of 82 patients because of failure after the first procedure. The remaining 70 patients were followed up. AVF patency was achieved in 63 patients (76.8%) in the 6^th^ month, 60 patients (73.1%) in the 12^th^ month, 53 patients (64.6%) in the 18^th^ month, and 44 patients (54%) in the 24^th^ month (Table 2). Factors (*Y*-axis variables in MDS), which were thought to affect the primary, secondary, and tertiary patency rates, including patient age, age of the fistula, AVF thrombosis, AVF stenosis, AVF aneurysm, AVF in the left or right arm, surgical embolectomy, diabetes, and hypertension, were investigated. Since no factors were detected significantly affecting the AVF patency (*X*-axis in MDS) in the evaluation performed on 82 patients; 70 patients, in whom the intervention was successful, were investigated. MDS results have been presented in Figures 8 and 9. The stress coefficient and *R*^2^ were used to evaluate the goodness of fit of the MDS solution. The stress coefficient and *R*^2^ values were found as 0.15 and 89.91%, respectively, showing that the MDS solution was a good choice for evaluating relations between the *X*-axis (patency) and the *Y*-axis (variables).

Figure 8 was drawn to find out similar and dissimilar patients in terms of all assessed characteristics. When Figure 8 was examined, it was seen that especially the patient numbers 50, 14, 55, and 3 were plotted separately in different locations on the chart. It can be considered that these findings indicate a different pattern of patient characteristics in these four patients compared to the rest of the study patients. When relations across the investigated patient characteristics were examined (Figure 9), it was observed that the major factors causing the differentiation of these patients were patient age and AVF age.

In the statistical analysis of the variables listed above, it was found that primary, secondary, and tertiary patency rates were not statistically different from each other. Then, tertiary patency rates were evaluated alone to define AVF patency.

When four patients (patient numbers 50, 14, 55, and 3), who were plotted distantly from the rest of the group, were examined, it was observed that fistula age of these patients (8, 18, 12, and 4 months, respectively) was notably below the mean value (37.24 months) as a common characteristic.

## 4. Discussion

In a retrospective study in the US in 2018, Arhuidese et al. evaluated and presented 476,926 patients with end-stage renal failure, who started haemodialysis treatment. Of the patients in that study, 81% started haemodialysis treatment with a catheter. The median maturation time was 47 days for autogenous fistulas and 29 days for prosthetic grafts (*P* < 0.001). An average of 0.1 interventions was performed per autogenous fistula and prosthetic graft before use (*P* < 0.001). The unadjusted Kaplan–Meier estimates of primary patency comparing autogenous fistulas to prosthetic grafts were 43.3% vs. 31.5% in the first year, 28.2% vs. 14.2% in three years, and 20.3% vs. 9.3% in five years, respectively. In that study, it was found that patients starting haemodialysis treatment with a catheter had low patency rates as well as high mortality and infection rates. On the contrary, patients who started haemodialysis treatment with an autogenous AVF achieved better patency and favourable quality of life with low infection rates [15]. That study clearly demonstrates that the use of catheters for haemodialysis poses a serious disadvantage for patients. Even if a new autogenous VA is created for a dysfunctional AVF, the maturation period can be as long as an average of 47 days. In our study, patients, in whom the thrill was detected after the procedure, underwent haemodialysis using the autogenous AVF on the day after the procedure or later without the need for a temporary dialysis catheter.

In the study conducted in 2000, Turmel-Rodrigues et al. performed 726 dilatation procedures, 135 stenting procedures, and 257 declotting procedures during a follow-up period of 12 years. One-year primary patency rates were found to be 50% for 209 consecutive forearm AVFs, 34% for 74 upper arm AVFs, and 25% for AVGs [16]. In the study conducted at three centres in Italy in 2019, Tozzi et al. performed a total of 311 angioplasties using drug-coated balloons on 200 patients. A total of 192 restenosis procedures were required in 81 patients during a mean follow-up period of 21 ± 8 months. Kaplan–Meier estimates indicated that 88.0%, 64.2%, and 40.6% of treated lesions were free from restenosis in the 6^th^, 12^th^, and 24^th^ months, respectively. A total of 51 (26%) lesions had been treated with conventional angioplasty within the previous 12 months. It was concluded in that study that fistuloplasty with DCB was advantageous for maintaining long-term AVF patency [17]. When we evaluated our clinical results, patency rates and the number of patients undergoing a successful procedure in the first intervention were 76.8% in 63 patients in the 6^th^ month, 73.1% in 60 patients in the 12^th^ month, 64.6% in 53 patients in the 18^th^ month, and 54% in 44 patients in the 24^th^ month. When these clinical results were compared with previous studies, it is seen that the clinical success rates were sufficient.

Two major reasons for native AVF failure are venous stenosis following maturation failure. Although the primary cause of the dysfunction of these fistulas is unclear, the characteristic lesion is a juxta-anastomotic stenosis. It is not known exactly whether the factor causing this stenosis is a venous constriction or venous neointimal hyperplasia. The stenosis occurs at or around the anastomotic region in wrist fistulas and in the proximal vein in fistulas created in the elbow area [16]. It has been shown that stenosed native AVFs contain muscle cells of venous neointimal hyperplasia expressing cytokines and mediators such as endothelin, platelet-derived growth factor (PDGF), and tumour growth factor (TGF-*α*); the latter has been shown to occur in the presence of oxidative stress markers. Causes of early failure in native AVFs are complex and multifactorial. Causative factors include a small artery (<1.5 to 2 mm) and a small vein (<2.0 to 2.5 mm), surgical manipulation and the use of a less-than-ideal technique, previous venepunctures, the development of accessory veins that divert blood away from the primary venous drainage conduit, haemodynamic stressors, and a possible genetic predisposition to vasoconstriction and neointimal hyperplasia after endothelial and smooth muscle injury. It is not known whether the maturation failure in early native AVFs is due to vascular constriction, neointimal hyperplasia, or a combination of both [18, 19]. At a clinical level, stenosis and thrombosis formation occur in dialysis access grafts and fistulas far more aggressively compared to arterial grafts or even interposed saphenous vein conduits in coronary artery bypass surgery potentially due to the following factors; first, at an anatomic level, veins tend to have a less well-defined internal elastic lamina, which could predispose to the migration of smooth muscle cells and myofibroblasts from the media to the intima. Second, at physiologic and molecular levels, veins tend to produce less nitric oxide (NO) and prostacyclin, which could predispose to endothelial injury [20]. Furthermore, many other factors including differences in gene expression in normal veins and arteries and puncture of dialysis grafts and fistulas could cause platelet thrombi and cytokine release. In addition, uraemia acting on the development of endothelial dysfunction and stenosis and difference in responses between arteries and veins to vascular injury may be involved in the development of venous neointimal hyperplasia [2].

In our study, when we have examined potential factors that might act on the patency rates after a successful balloon fistuloplasty procedure at the first attempt on 70 patients, we have found out that the major factors involved are patient age and the age of the fistula as the patient-associated and AVF-associated factors, respectively. Advanced age might favourably affect patency rates through mechanisms affecting the development of especially neointimal hyperplasia and stenosis. With advancing age, the release of some mediators involved in the development of neointimal hyperplasia and stenosis may decrease [21]. Furthermore, it is suggested that smooth muscle progenitor cells involved in the development of neointimal hyperplasia may originate from the bone marrow and that the reduction in bone marrow in advanced age may act on patency rates favourably in old individuals [22].

## 5. Conclusion

Restoration of the function of a dysfunctional native AVF is associated with a serious advantage for the patient. The patency rates in the 2 year follow-up of the patients undergoing a successful procedure at the first attempt in this study demonstrate that clinical success is sufficient. The MDS analysis has revealed that the following two factors are effective as patient-associated and native AVF-associated factors in achieving success, age of the AVF fistula and age of the patient, the latter being effective as much as 2.5 times compared to the former. Advanced age of the patient and the fistula may alleviate many factors involved in neointimal hyperplasia and stenosis formation. Balloon fistuloplasty of the draining vein is safe with low complication rates and is clinically effective, and it is associated with increasing success rates in advanced age and in cases with aged native AVFs.

## Figures and Tables

**Table 1:** Study population and vascular access characteristics.

Number of patients, *n* (%)	**82 (100%)**
Age (years)	Mean age 59.05 ± 9.3 (range 27–88)
Female, *n* (%)	32 (39.0%)
Male, *n* (%)	50 (60.9%)
Smokers, *n* (%)	8 (9.7%)
Peripheral arterial disease, *n* (%)	11 (13.4%)
Coronary artery disease, *n* (%)	8 (9.7%)
Hyperlipidaemia, *n* (%)	14 (17.0%)
Diabetes mellitus, *n* (%)	59 (71.9%)
Hypertension *n* (%)	59 (71.9%)
Native AVF location, *n* (%)	Radio-cephalic 55 (67.0%)
	Brachio-cephalic 24 (29.2%)
	Brachio-basalic 3 (3.6%)
Native AVF side, *n* (%)	Left arm 66 (80.4%)
	Right arm 16 (19.5%)
Pathological lesion of AVF, *n* (%)	AVF stenosis 39 (47.5%)
	AVF thrombosis 43 (52.4%)
	AVF aneurysm 28 (34.1%)
Location of pathological lesion, *n* (%)	Venous stenosis 46 (56.0%)
	Juxta-anastomotic stenosis 26 (31,7%)
	Anastomotic stenosis 8 (9.7%)
	Central vein stenosis 1 (1.2%)
	Arterial stenosis 1 (1.2%)
Age of AVF (months)	37.24 ± 5.6
Goodness of fit (variance accounted for)	0.8990167

**Table 2:** Distribution of rates of procedure success and patency by months.

	Primary patency *n* (%)	Secondary patency *n* (%)	Tertiary patency *n* (%)
Success of first endovascular intervention	70 (85%)	70 (85%)	70 (85%)
6th month patency	57 (69.5%)	63 (76.8%)	63 (76.8%)
12th month patency	46 (56%)	59 (71.9%)	60 (73.1%)
18th month patency	39 (48%)	51 (62.1%)	53 (64.6%)
24th month patency	32 (39%)	41 (50%)	44 (54%)

## Data Availability

The data used to support the findings can be found in digital recordings of Elazığ Fethi sekin City Hospital

## Additional Points

Balloon fistuloplasty from drainage vein is safety, easy to apply, and a successful method. Advanced patient age and AVF age are positively affecting factors of the method results. Heamodialysis from native AVF presents better renal replacement therapy and quality of life.
